# A bibliometric analysis of research on dementia comorbid with depression from 2005 to 2024

**DOI:** 10.3389/fnins.2025.1508662

**Published:** 2025-02-04

**Authors:** Xia Li, Wei Su, Lili Cai

**Affiliations:** Department of Psychiatry, Huzhou Third Municipal Hospital, the Affiliated Hospital of Huzhou University, Huzhou, China

**Keywords:** dementia, depression, bibliometric, global trends, mechanism

## Abstract

**Introduction:**

With the global rise in life expectancy, the incidence of dementia is increasing, often accompanied by depressive symptoms. Understanding the interplay between dementia and depression is crucial, as depression may not only co-occur with but also potentially exacerbate the progression of dementia. This study employs bibliometric analysis to map the global research landscape, identify prevailing themes, and discern future research directions.

**Methods:**

We analyzed reviews and original research articles on dementia and depression extracted from the Web of Science Core Collection spanning from 2005 to 2024. Utilizing tools such as CiteSpace, VOSviewer, and an R-based bibliometric analysis package, we assessed trends in publication volume, citation frequency, contributing countries, leading institutions, predominant journals, influential authors, and emergent keywords.

**Results:**

A total of 1972 publications were obtained, revealing a consistent increase in both the number of publications and their citation impact over the study period. The United States is the country with the most publications and the most extensive collaborations. The University of Toronto and the Journal of Alzheimer’s Disease were identified as key contributors to this field. This research area is currently focused on cognitive impairments, the role of gut microbiota, and non-drug interventions. Future directions emphasize the importance of early detection and intervention, a deeper understanding of the gut-brain axis, and the integration of technology in treatment strategies. Additionally, there is a growing interest in the physiological and psychological interplays such as oxidative stress and its implications.

**Conclusion:**

This study underscores pathogenesis, comorbid conditions, and non-drug interventions as primary research focal points, suggesting these areas as potential pathways for therapeutic innovation. These insights are intended to deepen our understanding, enhance diagnostics, and improve the management of dementia and depression, providing guidance for future research aimed at addressing these escalating global health challenges.

## 1 Introduction

Dementia is a chronic and progressive disease that severely affects memory, intelligence, thinking ability, and social skills (Weiner and Lipton, 2009). The primary types are Alzheimer’s disease (AD) and vascular dementia (Goodman et al., 2017). The global prevalence of dementia is expected to rise from approximately 57.4 million cases in 2019 to 152.8 million by 2050 (GBD 2019 Dementia Forecasting Collaborators, 2022). Study indicates a trend toward earlier onset of dementia (Ryan et al., 2022). Depression, an affective disorder, is characterized by low mood, decreased interest, and slowed thinking (Smith, 2014), affecting over 300 million individuals worldwide (World Health Organization, 2017). Depression is debilitating and results in a diminished quality of life (GBD 2017 Disease and Injury Incidence and Prevalence Collaborators, 2018; Bromet et al., 2011). Both dementia and depression not only profoundly affect cognitive and emotional state but also impose significant burdens on social and economic systems. It is estimated that dementia was costing the global economy US$ 818 billion in 2015, which is equivalent to 1.1% of the world’s GDP (Alzheimer's Disease International, n.d.). Meanwhile, depression is ranked by the World Health Organization as the third leading cause of the global burden of disease (World Health Organization, 2021).

Research indicates that a significant proportion of individuals with dementia also experience depressive symptoms; approximately 38–40% present with these symptoms, while 16% are diagnosed with major depressive disorder (MDD) (Leung et al., 2021; Goodarzi et al., 2017). Concurrently, chronic depressive states may increase the likelihood of dementia (Cantón-Habas et al., 2020). The connection between dementia and depression is evident at the molecular and cellular levels, where neurodegeneration, neuroinflammation, and neurotransmitter imbalances serve as common pathological substrates. A notable finding is that amyloid-*β* (Aβ) deposition—a hallmark of dementia—is associated with an increased prevalence of depressive symptoms among cognitively normal older adults, suggesting that such symptoms could be early indicators of dementia (Donovan et al., 2018). Depression is further characterized by a dysregulation of the hypothalamic–pituitary–adrenal (HPA) axis, which leads to elevated glucocorticoid secretion (Hermida et al., 2012). This increase can cause structural and functional impairments in the hippocampus, crucial for memory and cognition (Frodl and O’Keane, 2013; Sudheimer et al., 2014), thereby providing biological evidence that depression may accelerate the progression of dementia.

Bibliometrics is widely used in the medical field as a common research methodology (Kokol et al., 2021). By analyzing the literature, bibliometrics provides a visual, comprehensive, and systematic elucidation of the development of a particular field, identifying current research hotspots and future trends (Chen et al., 2012). A bibliometric study of behavioral and psychological symptoms of dementia, published in 2023, found that depression was ranked in the top 10 keywords (Cai et al., 2024). Furthermore, analysis from a bibliometric study on late-life depression predicts that dementia will be a hot topic for future research (Du et al., 2024). Studying of the interaction between dementia and depression will not only elucidate the mechanisms of comorbidity in these diseases but also assist physicians in more effectively managing these common and complex conditions in their clinical practice.

This study employs bibliometrics to analyze the information of countries, regions, authors, and journals, aiding researchers in identifying potential collaborators and selecting appropriate journals for submissions. Additionally, by analyzing keywords and highly cited papers, the study investigates current research trends and key focus areas in the fields of dementia and depression. This comprehensive analysis provides invaluable information, serving as a crucial guide for future research directions and the development of effective treatment strategies.

## 2 Methods

### 2.1 Data collection

**Figure 1:**
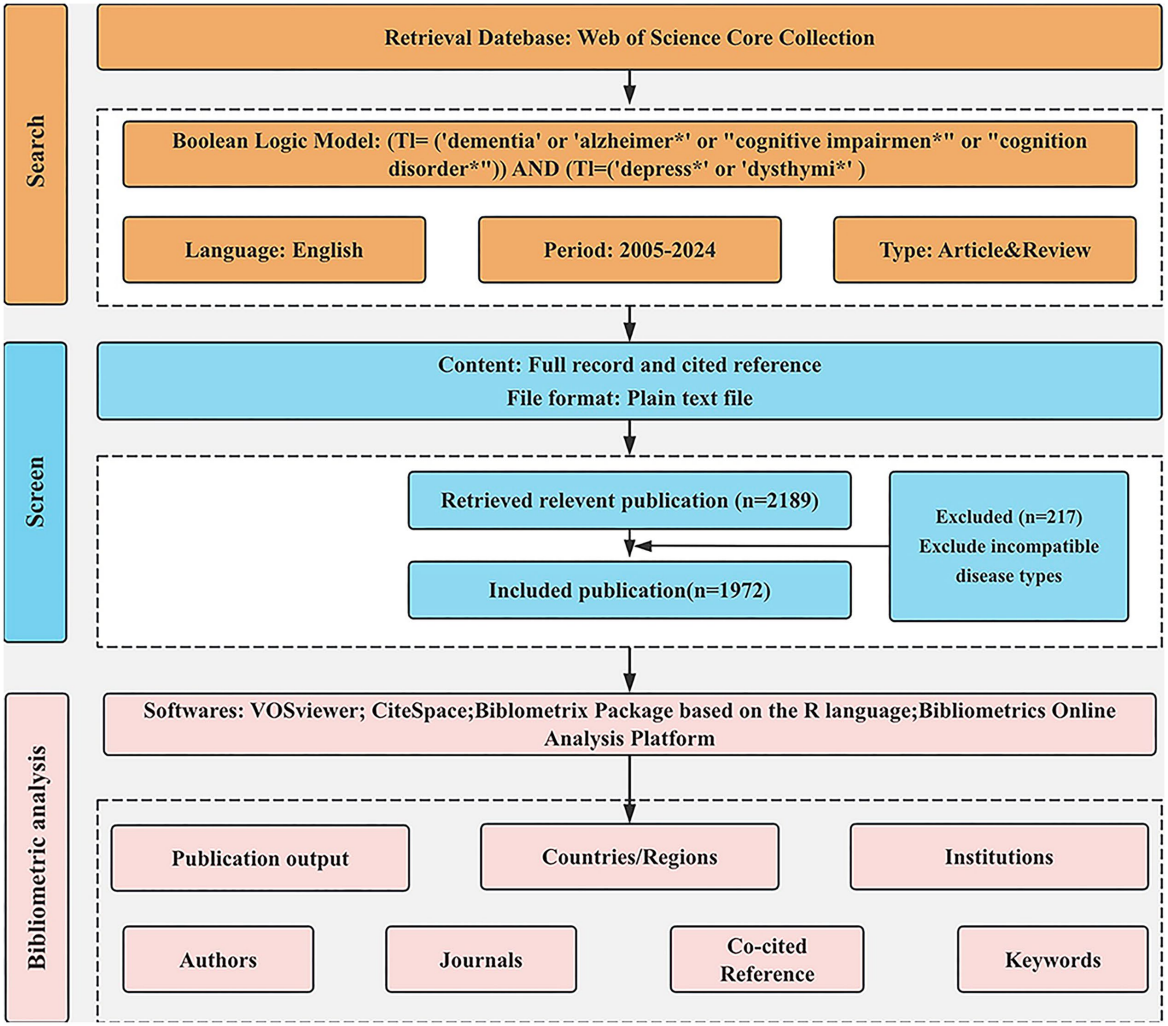
The detailed retrieval process.

The data relevant to dementia and depression for this study were obtained from the Web of Science Core Collection (WoSCC). WoSCC is the best known and most influential scientific citation database used for bibliometric analysis. The search strategy is as follows: TI = [(‘dementia’ OR ‘Alzheimer∗’ OR ‘cognition disorder*’ OR ‘cognitive impairment*’) AND (‘depress∗’ OR ‘dysthymi*’)]. The search was conducted over a period from June 19, 2005, to June 19, 2024. A total of 3,390 documents were obtained. We restricted our focus to English-language publications categorized as “reviews” or “articles.” 1,201 articles of other types were excluded, including Proceeding Paper (*n* = 51), Retracted Publication (*n* = 2), Letter (*n* = 66), Meeting Abstract (n = 794), Editorial Material (*n* = 99), Correction (*n* = 32), News Item (*n* = 21), Early Access (*n* = 11), Book Chapters (*n* = 36), Book (*n* = 1), Book Review (*n* = 1), Retraction (*n* = 1), Non-English (*n* = 86). A total of 2,189 publications were initially retrieved. Papers that were irrelevant to the research topic were systematically excluded after reviewing titles, abstracts, and, in some cases, the full texts. We excluded articles where the subjects were spouses or caregivers of people with dementia. Finally, 1,972 papers were deemed relevant and included in the analysis. There are 1,697 articles and 275 reviews. The search process is shown in Figure 1.

This study did not involve any animal or human subjects; therefore, no ethical approval was required.

### 2.2 Data analysis and tools

VOSviewer 1.6.20 and CiteSpace 6.3.R1 were used for bibliometric analysis in this study. VOSviewer is one of the most widely used tools for quantitative analysis in bibliometrics. It provides a systematic understanding of the structure and dynamic development of scientific research (Van Eck and Waltman, 2010). The VOSviewer tool is employed to visualize highly productive countries, institutions, journals, and authors, as well as highly cited journals and authors, thereby enhancing the understanding of relevant research.

CiteSpace, a Java-based visual bibliometric analysis software developed by Chen and Song (2019) and Chen (2006), visualizes research field hotspots and evolution, predicting developments to some extent. Co-occurrence analysis can reveal the focal areas of research. Additionally, CiteSpace uses cluster analysis to group co-cited references and keywords, further unveiling the relationships and structures between different research topics. Its burst detection feature is able to identify keywords that experience a sharp increase in citations within a specific time window, which aids in predicting research trends and identifying emerging research hotspots. And Microsoft Office Excel 16.84 is used to analyze annual publication numbers and related citation trends.

Bibliometric online analysis platform^1^ to analyze international cooperation between countries. Bibliometrix is an online visualization software package based on R language, which can perform statistical analysis on literature, calculate indexes, and present research hotspots.

## 3 Results

### 3.1 Annual outputs and citation trends

**Figure 2:**
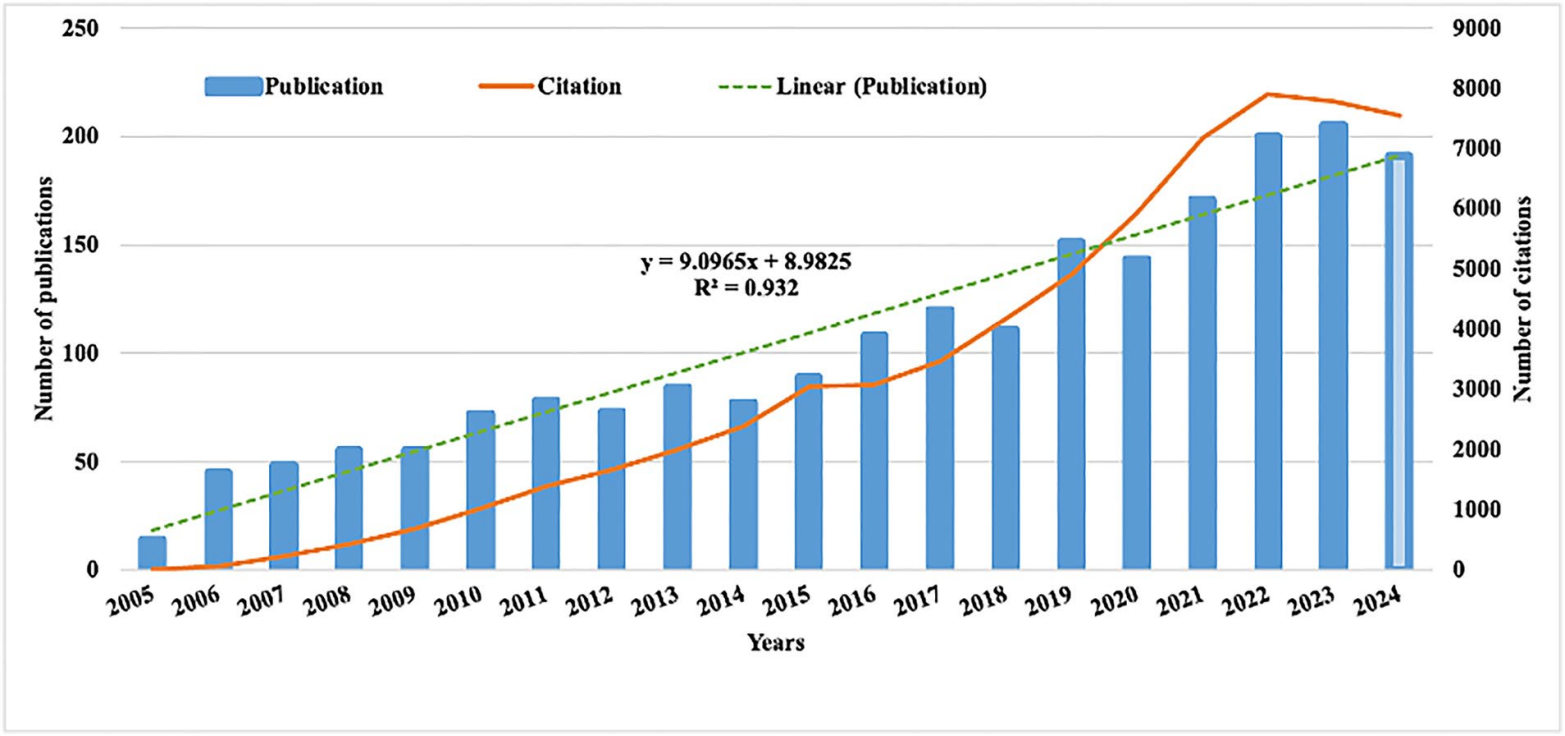
Annual publication and citation.

The number of publications and their related citations serve as key indicators to visualize research trends. Over the past 20 years, the volume of publications concerning dementia and depression has steadily increased (Figure 2). As of June 19, 2024, a total of 1,972 articles and reviews have been published, underscoring the continuing significance of this research area. This trend is mirrored in the increasing number of citations, reflecting the growing influence and ongoing relevance of this research area. Using Excel software, a predictive growth model was developed. By the year 2030, it is projected that 236 new articles will be published in this field.

### 3.2 Most contributing countries/regions and institutions

**Figure 3:**
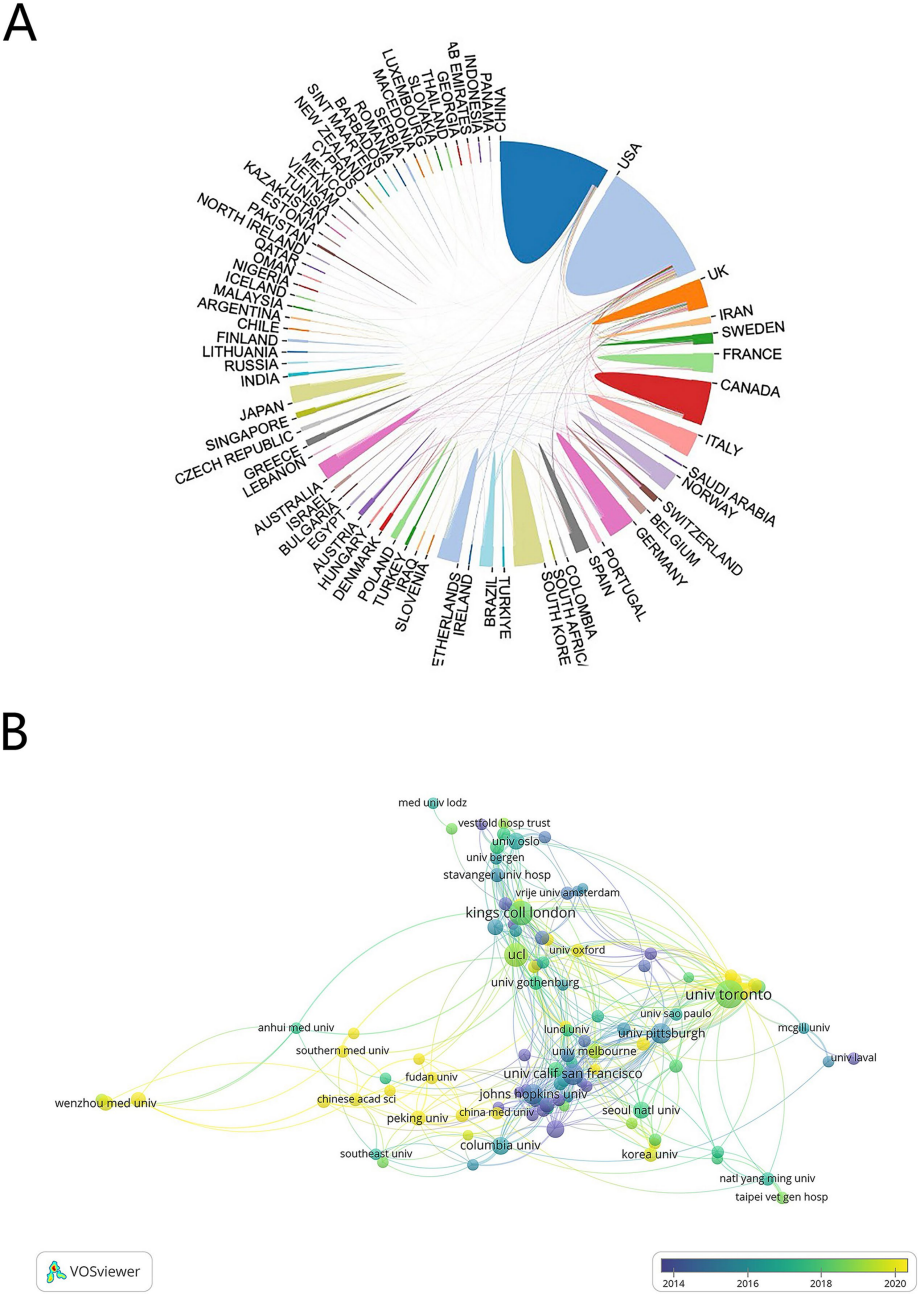
**(A)** Country cooperation network map. **(B)** Visualization of institutional cooperation.

A total of 81 countries and regions participated in this area, with 27 of these contributing 20 or more publications each. The USA leads with 514 publications (22,097 citations), followed by China with 313 publications (4,733 citations) (Table 1). An international cooperation network map (Figure 3A) reveals collaborative relationships among 70 of the 81 countries, with different colors marking each on the map. The USA is the most frequent collaborator, followed by the United Kingdom and Germany, though China, despite its high publication count, shows fewer international collaborations. On the institutional level, 2,658 organizations engage in this research, with 104 having published 10 or more works. The University of Toronto, with 68 publications, leads, followed by King’s College London and University College London with 52 and 46 publications, respectively. Four of the top 10 institutions are in the USA, collectively publishing 129 papers (Table 2). Figure 3B shows the temporal distribution of publications, highlighting the University of Toronto’s significant output and extensive collaborations from 2018 to 2020, with yellow indicating recent activity.

**Table 1:** Top 10 countries in terms of number of publications.

Rank	Countries	Counts	Citations	TLS
1	USA	514	22,097	279
2	China	313	4,733	91
3	England	169	6,456	226
4	Germany	132	3,494	153
5	Canada	131	4,976	116
6	South Korea	117	1734	46
7	Australia	106	4,602	115
8	Netherlands	103	5,242	117
9	Italy	99	3,530	71
10	Japan	84	1705	34

**Table 2:** Top 10 organizations in terms of publications.

Rank	Institutions	Counts	Citations	TLS
1	University of Toronto (Canada)	68	2,512	167
2	King’s College London (England)	52	1,573	132
3	University College London (England)	46	1867	57
4	University of California, San Francisco (UAS)	39	3,044	98
5	University of Pittsburgh (USA)	36	2,595	97
6	Johns Hopkins University (India)	34	1,153	57
7	University Health Network (Canada)	27	798	97
8	Columbia University (USA)	27	1,638	42
9	Duke University (USA)	27	1,263	36
10	University of Oslo (Norway)	25	602	57

### 3.3 Analysis of author

**Figure 4:**
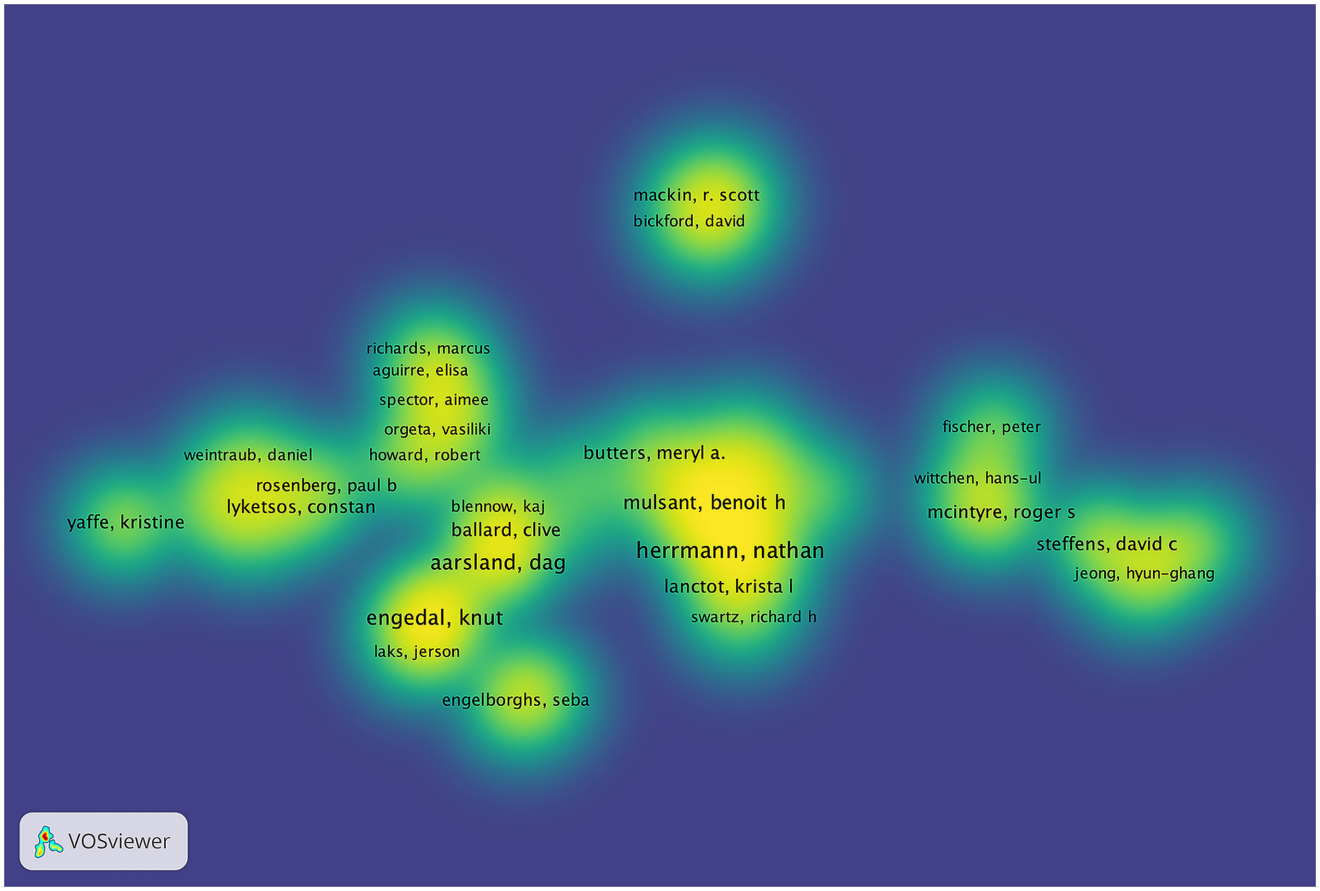
The author’s density visualization.

**Figure 5:**
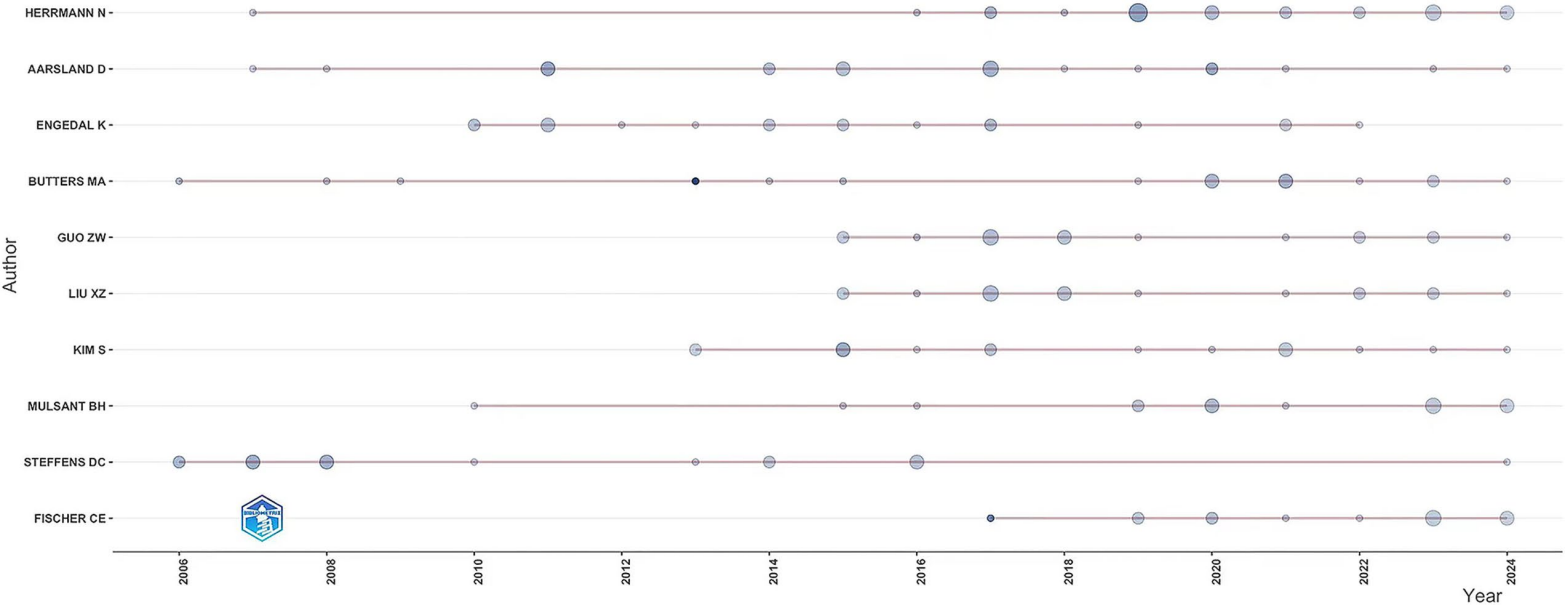
The line represents an author’s timeline. The bubble size is proportional to the n (n is year) of documents. The color intensity is proportional to the total citation per year.

The study identified 9,570 authors publishing on dementia and depression, with Herrmann N (Hirsch, 2005), Aarsland D (Du et al., 2024), and Engedal K (Kokol et al., 2021) leading (Table 3). Aarsland D has the highest H-index, indicating significant citation impact (Hirsch, 2005; Hirsch, 2007). The G-index and M-index further reflect the authors’ scholarly impact (Ali, 2021). A central cooperative network among these top contributors is evident in the author density visualization (Figure 4). Notably, Engedal K has ceased publishing since 2022, while Herrmann N remains highly active and cited (Figure 5).

**Table 3:** Top 10 authors in terms of publications.

Author	NP	H_index	G_index	M_index	TC	PY _start
Herrmann N	25	12	23	0.667	572	2007
Aarsland D	21	14	21	0.778	795	2007
Engedal K	18	12	18	0.8	473	2010
Butters MA	17	11	17	0.579	1,527	2006
Guo ZW	17	9	15	0.9	244	2015
Liu XZ	17	9	15	0.9	244	2015
Steffens DC	16	13	16	0.684	999	2006
Kims	16	9	16	0.75	326	2013
Mulsant BH	16	9	14	0.6	202	2010
Lyketsos CG	14	13	14	0.684	743	2006

### 3.4 Analysis of journals and co-cited journals

A total of 605 journals have published papers on dementia and depression. Table 4 shows that the top 10 journals, primarily in the fields of geriatric psychiatry and neurology, include the *Journal of Alzheimer’s Disease*, which leads with 100 publications. It is closely followed by the *International Journal of Geriatric Psychiatry* and the *American Journal of Geriatric Psychiatry*, with 96 and 86 publications, respectively. The influence of each journal is gauged by the number of co-citations it receives. The more co-citations, the greater the journal’s impact. *Neurology* ranks as the most cited journal, with 3,092 citations, highlighting its significant academic value in neurology. The *International Journal of Geriatric Psychiatry* and *American Journal of Geriatric Psychiatry* are also highly cited, with 3,067 and 2,993 citations, respectively.

**Table 4:** Top 10 journals and co-cited journals.

Rank	Journals	Count	IF	JCR	Rank	Co-cited journals	Citations	IF	JCR
1	Journal of Alzheimer’s Disease	100	3.4	Q2	1	Neurology	3,092	7.7	Q1
2	International Journal of Geriatric Psychiatry	96	3.6	Q1	2	International Journal of Geriatric Psychiatry	3,067	3.6	Q1
3	American Journal of Geriatric Psychiatry	86	4.4	Q1	3	American Journal of Geriatric Psychiatry	2,993	4.4	Q1
4	Journal of Affective Disorders	78	4.9	Q1	4	American Journal of Psychiatry	2,240	15.1	Q1
5	International Psychogeriatrics	52	4.6	Q1	5	Journal of Affective Disorders	2,192	4.9	Q1
6	Aging & Mental Health	34	2.8	Q2	6	Archives of General Psychiatry	2,189		
7	Journal of Geriatric Psychiatry and Neurology	34	2.9	Q2	7	Biological Psychiatry	2,011	9.6	Q1
8	Frontiers in Aging Neuroscience	30	4.1	Q2	8	Journal of Alzheimer’s Disease	1,730	3.4	Q2
9	Dementia and Geriatric Cognitive Disorders	29	2.2	Q3	9	Journal of the American Geriatrics Society	1,501	4.3	Q1
10	Journal of Alzheimer’s Disease	100	3.4	Q2	1	Neurology	3,092	7.7	Q1

### 3.5 Analysis of keywords

#### 3.5.1 Keyword co-occurrence

**Figure 6:**
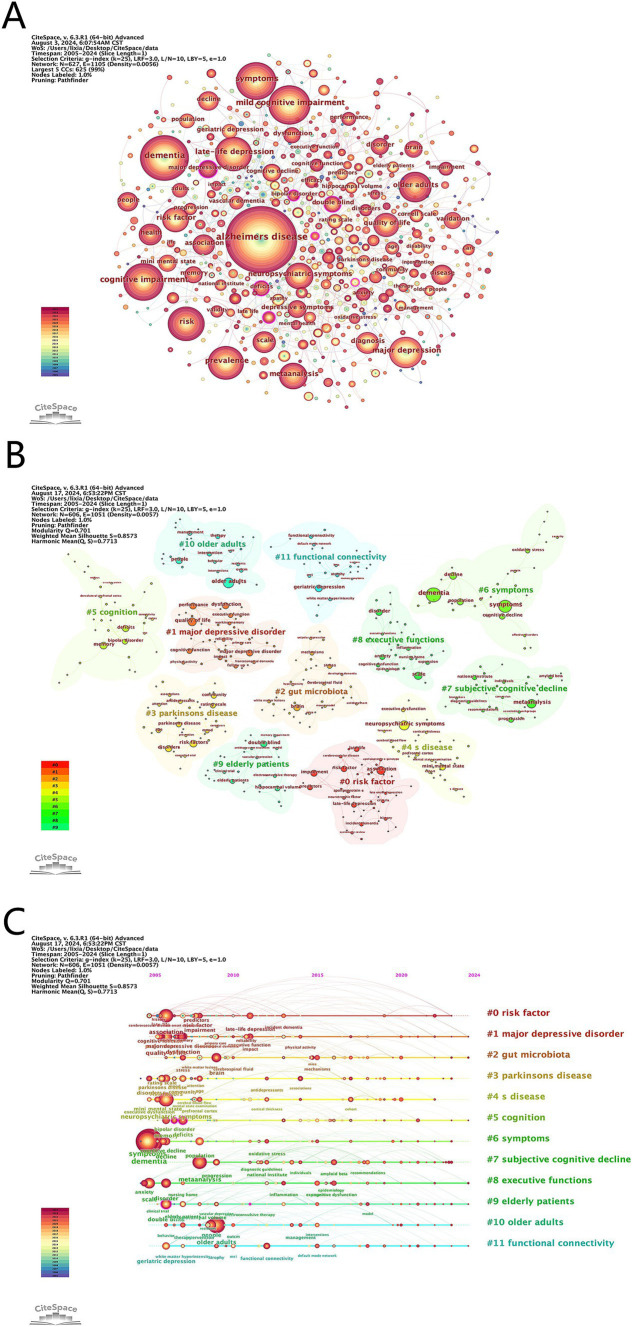
**(A)** Visualization of keywords. **(B)** Cluster analysis of keywords. **(C)** Timeline graph of cluster analysis.

We deleted and merged keywords. The merged keywords are synonyms, such as merging Alzheimer disease into Alzheimer’s disease and late-life depression into late life depression. The deleted keywords are meaningless keywords that cannot provide key information, such as patient and life. Table 5 highlights the top 10 keywords ranked by frequency in this field. A visual map of the keywords was shown in Figure 6A. The frequent keywords Alzheimer’s disease, dementia, and mild cognitive impairment (MCI) emphasize the research focus on the link between depression and dementia. This highlights ongoing efforts to understand how depressive disorders interact with cognitive declines, aiming to identify diagnostic markers and therapeutic targets for these coexisting conditions. Centrality highlights key concepts in the research network. The top three keywords—bipolar disorder (0.15), electroconvulsive therapy (0.12), and double blind (0.12)—underscore the importance of rigorous study design, the link between bipolar disorder and dementia, and treatment of patients with dementia and depression.

**Table 5:** Top 10 keywords by frequency and centrality.

Rank	Keywords	Frequency	Rank	Keywords	Centrality
1	Alzheimer disease	942	1	Bipolar disorder	0.15
2	Dementia	495	2	Electroconvulsive therapy	0.12
3	Mild cognitive impairment	374	3	Double blind	0.12
4	Cognitive impairment	348	4	Deficits	0.11
5	Prevalence	331	5	Mood disorder	0.11
6	Risk	300	6	Major depressive disorder	0.10
7	Late-life depression	287	7	Anxiety	0.10
8	Major depression	273	8	Association	0.09
9	Older adults	233	9	Cognitive decline	0.09
10	Neuropsychiatric symptoms	201	10	Intervention	0.09

#### 3.5.2 Keyword clustering timeline

We performed a cluster analysis of the keywords (Figure 6B) to identify trending topics in the field. Modularity Q and Mean Silhouette (S) are crucial indicators for evaluating the validity of a clustered map. A Q value greater than 0.3 indicates a significant clustering structure, while an S value greater than 0.7 suggests convincing clustering. In this study, the S value is 0.86 and the Q value is 0.71, indicating reliable clustering results. Figure 6C presents a timeline of the keywords, highlighting the research progress of keyword nodes in each cluster. The clustering results reveal that #2 gut microbiota, #8 executive functions and #11 functional connectivity are current research hotspots. These clusters reflect an interest in how gut health affects neurological conditions, the cognitive deficits common in both disorders, and the brain network changes linking depression and cognitive impairments.

**Figure 7:**
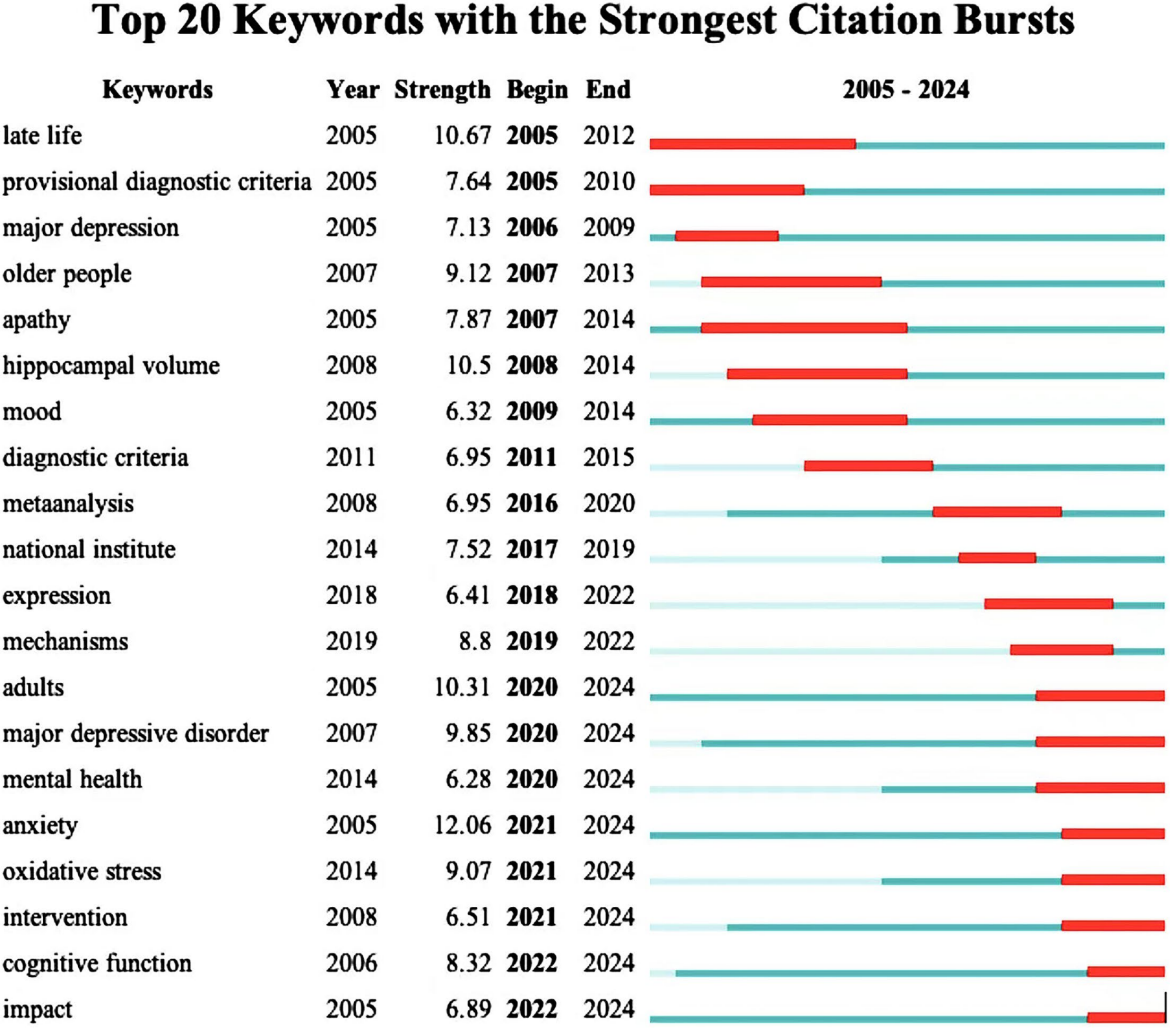
Top 25 keywords with the strongest bursts.

The citation burst analysis reveals evolving trends in dementia and depression research (Figure 7). Early (2005–2011) focus areas included “late life,” “major depression” and “hippocampus volume,” while mid-period (2012–2019) research emphasized “diagnostic criteria,”” meta-analysis “and “mechanisms.” Recent (2020–2024) hotspots highlight “oxidative stress,” “anxiety,” “cognitive function,” and “intervention,” indicating current cutting-edge interests. Overall, the study highlights a shift towards understanding oxidative stress, anxiety, and cognitive aspects in dementia and depression.

**Figure 8:**
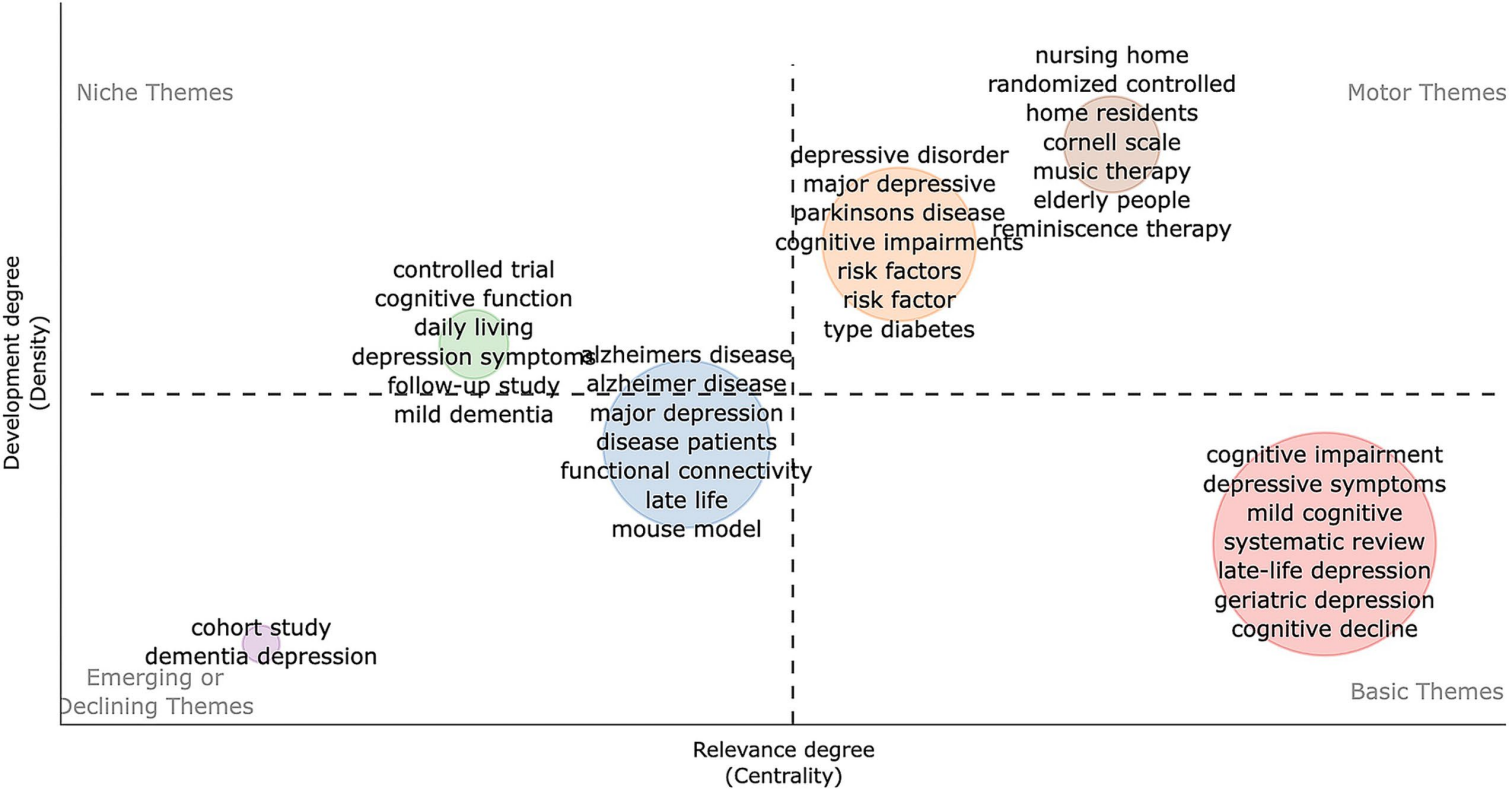
Development trend and importance of hot spots. The X-axis represents the centrality indicating the importance of a theme; The Y-axis symbolizes the density indicating the development of a theme.

The keyword quadrant map (Figure 8) highlights Motor Themes such as “nursing home,” “randomized controlled,” “home residents,” “music therapy” and “reminiscence therapy,” showing they are central and well-developed areas focusing on elderly care interventions. Basic Themes like “cognitive impairment” and “depressive symptoms” are crucial but need further development. Niche and Emerging/Declining Themes indicate specialized and less-attended areas. In conclusion, trends point to early detection of cognitive decline, the impact of gut health on cognition, and managing dementia and depression in elderly care.

### 3.6 Analysis of co-cited references

**Figure 9:**
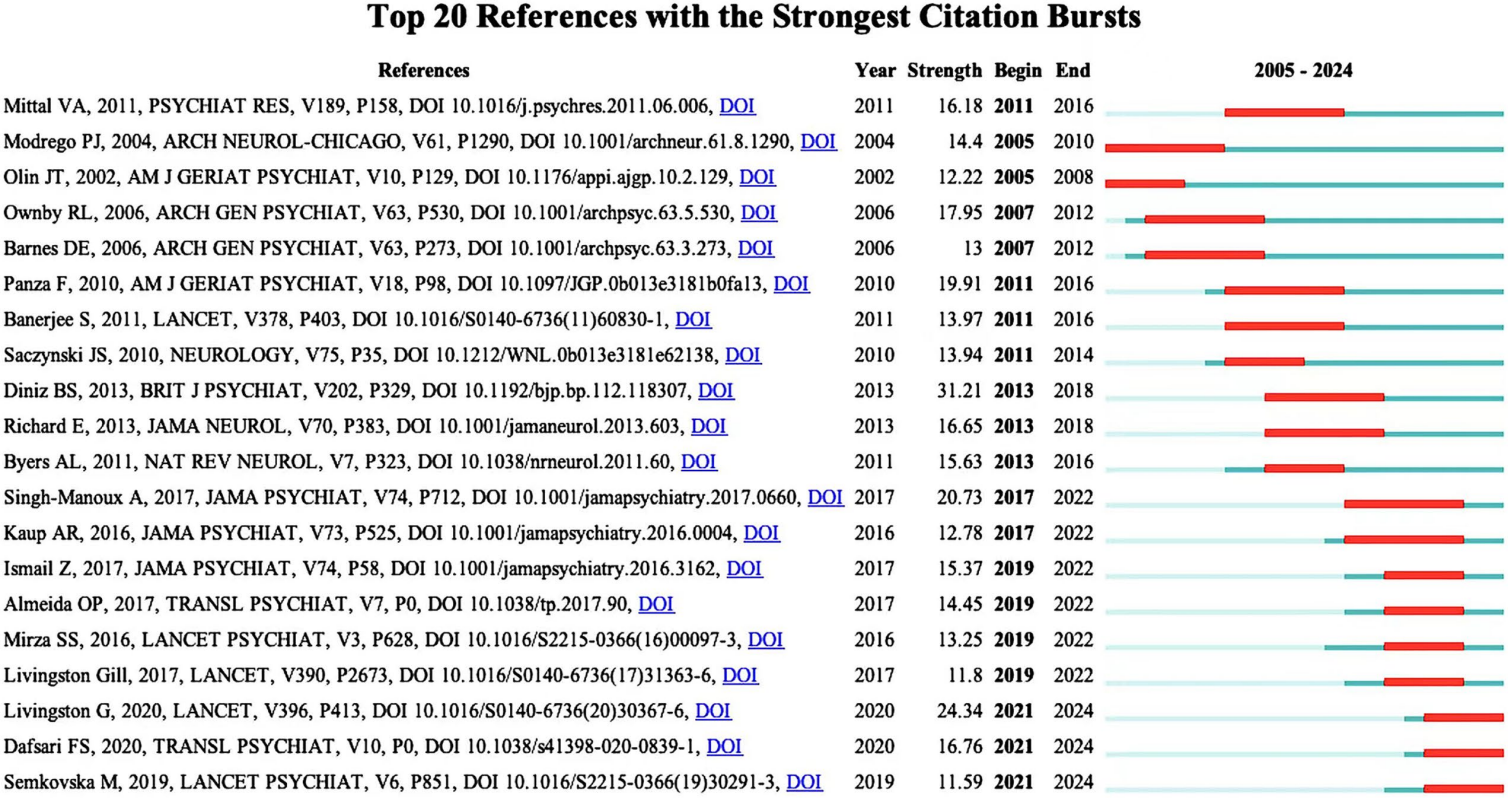
Top 25 reference with the strongest bursts.

Table 6 lists the top 10 most frequently co-cited literature. They have high academic value and influence. It can be used by new researchers to get a quick overview of the field. Figure 9 shows the top 20 References with the Strongest Citation Bursts. Recent citation bursts between 2021 and 2024 highlight three key publications in *The Lancet* and *Translational Psychiatry*. Livingston G’s 2020 study discusses management strategies for depression and dementia. Dafsari FS explores links between antidepressant treatment and prevention of dementia in 2020. Semkovska M’s 2019 paper focuses on effects of depression remission on cognition. These studies, significant in the fields of depression and dementia, have garnered substantial attention for advancing understanding and treatment approaches.

**Table 6:** Top 10 highly cited literature.

Rank	Citation	Author	Title	Journal	Year
1	71	Diniz BS	Late-life depression and risk of vascular dementia and Alzheimer’s disease: systematic review and meta-analysis of community-based cohort studies	BRIT J PSYCHIAT	2013
2	54	Singh-Manoux A,	Trajectories of Depressive Symptoms Before Diagnosis of Dementia: A 28-Year Follow-up Study	JAMA PSYCHIAT	2017
3	52	Livingston G	Dementia prevention, intervention, and care: 2020 report of the Lancet Commission	LANCET	2020
4	43	Panza F	Late-life depression, mild cognitive impairment, and dementia: possible continuum?	AM J GERIAT PSYCHIAT	2010
5	41	Ismail Z	Prevalence of Depression in Patients with Mild Cognitive Impairment: A Systematic Review and Meta-analysis	JAMA PSYCHIAT	2017
6	38	Richard E	Late-life depression, mild cognitive impairment, and dementia	JAMA NEUROL	2013
7	37	Ownby RL	Depression and risk for Alzheimer disease: systematic review, meta-analysis, and metaregression analysis	ARCH GEN PSYCHIAT	2006
8	36	Dafsari FS	Depression-an under recognized target for prevention of dementia in Alzheimer’s disease	TRANSL PSYCHIAT	2020
9	33	Byers AL	Depression and risk of developing dementia	NAT REV NEUROL	2011
10	33	Almeida OP	Depression as a modifiable factor to decrease the risk of dementia	TRANSL PSYCHIAT	2017

## 4 Discussion

### 4.1 General information

In this study, we conducted a comprehensive bibliometric analysis of 1972 publications related to dementia and depression published on WoSCC from 2005 to 2024. From 2005 to 2024, the growing number of publications on dementia and depression underscores an expanding research focus and increasing awareness of these critical public health issues. The growing number of publications on dementia and depression can be attributed to several key factors. First, the aging of the global population has exacerbated the prevalence of dementia and depressive diseases, thereby increasing research interest among researchers. The United Nations Population Division released data in July 2024. By the end of the 2070s, the global population aged 65 and over is expected to reach 2.2 billion, exceeding the number of children under the age of 18, which will exacerbate social and economic impacts. Second, advances in diagnostic techniques such as neuroimaging and biomarkers enable early detection of dementia and its early depressive symptoms. Aggrephay-related gense (ARGs) can also be used to identify new diagnostic markers for AD through machine learning methods (Xu et al., 2023). This has stimulated research into their common pathologies and potential treatments. Third, increasing awareness of the critical role of mental health has fueled research into depression and dementia. In addition, there has been a significant increase in research into non-pharmacological interventions, including cognitive training and physical activity, which are becoming complementary to traditional pharmacological treatments. The field demonstrates robust global engagement, with the USA leading in publication volume followed by China. The USA also leads in research impact, evidenced by high citation and collaboration rates. China needs to improve the quality of its publications and international collaborations. The close collaborations, especially between the USA and China, emphasize the field’s global scope and the importance of international partnerships in fostering advancements. In recent years, some countries have made outstanding contributions in the field of dementia and depression. In 2023, a Norwegian scholar published an article in Lancet Diabetes Endocrino that systematically sorted out the effects of steroid hormones, especially estrogen, on the female brain and its impact on the risk of depression and dementia. This furthered our understanding of the complex interactions of risk and protective factors on female brain health and provided a neurological framework for vulnerability and resilience (Barth et al., 2023). In addition, a randomized clinical trial study published in JAMA Psychiatry by Canadian researchers in 2024 found that cognitive remediation and transcranial direct current stimulation, both targeting the prefrontal cortex, can effectively slow the risk of cognitive decline in those with remitted MDD (with or without MCI) and those with a low genetic risk of Alzheimer’s disease (Rajji et al., 2024). Analyzing each international contribution to dementia and depression research will help identify key research strengths and potential international partnerships.

The University of Toronto stands out among the top ten research institutions, with Herrmann N as its leading author. The 2023 publication by Herrmann N in *Biological Psychiatry* investigates the integrity of gray and white matter in cases of remitted major depressive disorder (rMDD), MCI, and concurrent rMDD and MCI. The study indicates that early-onset, treated MDD might not induce structural changes linked to cognitive decline (Marawi et al., 2023). Herrmann’s highly cited 2018 study of 1,965 participants with depression and MCI found that the amnestic MCI subtype and active depression within the past 2 years independently increased the risk of AD dementia (Gallagher et al., 2018). Another influential study reported that MCI patients with apathy alone or both apathy and depression had a higher risk of progressing to AD compared to those without neuropsychiatric symptoms (Ruthirakuhan et al., 2019). Furthermore, Aarsland D, who boasts the highest H-index, along with his colleagues, discovered an association between late-life depression and abnormal amyloid protein metabolism by comparing synaptic function markers and AD pathology in patients with geriatric depression, pre-dementia AD, and a control group (Siafarikas et al., 2021). In a separate study in 2022, Engedal K used magnetic resonance imaging to compare patients with dementia against those with both dementia and depressive symptoms, noting distinct differences in the morphology of the temporal lobe—specifically, reduced sizes in the left temporal pole and right transverse temporal cortex in the latter group (Barca et al., 2022). Research on dementia and depression is predominantly published in Q1 and Q2 geriatric psychiatry and neurology journals, underscoring the high quality and credibility of the studies. The Journal of Alzheimer’s Disease leads in publications on Alzheimer’s etiology, pathogenesis, and treatment, while Neurology, sponsored by the American Academy of Neurology, is the most cited, reflecting its prestige and quality.

### 4.2 Research basic

High-frequency keywords in co-occurrence analyses reflect the research hotspots within a field. In this study, the top three keywords are AD, dementia, and MCI. AD is the main type of dementia, accounting for approximately 60–80% of all dementia (Yepes, 2021). According to the latest clinical staging guidelines for AD, the development of dementia is divided into 7 stages. Stage 0 refers to carrying the familial AD pathogenic gene; stage 1 is the discovery of positive core markers of AD; stage 2 is characterized by subjective cognitive decline; stage 3 is mild cognitive impairment; stage 4 is mild dementia; stage 5 is moderate dementia; stage 6 is severe dementia (Jack et al., 2024).

Early detection and intervention of MCI are crucial to delaying the progression of dementia (Ganguli et al., 2004). Depression is a common psychiatric symptom in patients with MCI and may signal the transition from MCI to dementia (Asmer et al., 2018; Cooper et al., 2015). Seoyoung Yoon demonstrated that the cognitive impairment in MCI patients with active depression was more pronounced than in those without depression, based on a one-year follow-up (Yoon et al., 2017). Meanwhile, Brooks et al. (2024) found that theta phase-gamma amplitude coupling (TGC) can distinguish individuals with MCI or MCI with rMDD (MCI + rMDD). In contrast, another study reported no significant in general cognition, as measured by Mini-Mental State Examination (MMSE), between MCI + rMDD and MCI patients (Duffy et al., 2014). The increased risk of cognitive decline in MCI patients with concurrent major depression compared to those with MCI alone remains uncertain and warrants further investigation.

The complex interplay between depression and dementia has been extensively studied, with particular attention to the temporal relationship between these conditions. The review published in 2013 from *The British Journal of Psychiatry* by Diniz et al. (2013) holds the highest citation count, revealing that late-life depression notably increases the risk of dementia, particularly vascular dementia and AD, through a comprehensive meta-analysis of 23 studies. Generally speaking, patients with depression in their later years have a poorer overall health condition, especially with a higher risk of cardiovascular and cerebrovascular diseases (Charlson and Peterson, 2002; Sheline et al., 2010). In addition, the co-occurrence of vascular diseases and AD suggests that many diagnoses of AD may actually represent mixed dementia cases (Laukka et al., 2010; Gold et al., 2007).

Further exploring the relationship between depression and dementia, a 28-year follow-up study involving 10,308 individuals by Singh-Manoux et al. (2017) a revealed that while depression in middle age does not increase the risk of dementia, but depressive symptoms in late life do. This finding suggests that these symptoms could be early indicators of dementia or have a common underlying cause, challenging the hypothesis that depression directly increases dementia risk (Singh-Manoux et al., 2017).

Enhancing the current understanding, the 2020 Lancet Commission report accentuates depression’s critical role in dementia progression (Livingston et al., 2020). The report refers to a pivotal study showing that the administration of selective serotonin-reuptake inhibitors (SSRIs) for over 4 years can delay the clinical manifestation of AD symptoms, which invites further exploration into whether antidepressants could lower the risk of dementia (Bartels et al., 2018). Together, these findings highlight the necessity of preventing, diagnosing early, and actively managing late-life depression not only to improve overall health but also to potentially decrease the incidence of dementia. The compelling evidence calls for additional research to determine how effective management of depression could delay or modify the course of dementia.

In conclusion, while late-life depression is consistently associated with an increased risk of dementia, its role as a precursor, comorbidity, or risk factor remains complex and multifaceted. The current evidence underscores the importance of early identification and effective management of late-life depression, not only to enhance overall health outcomes but also to potentially delay or modify the trajectory of dementia. Future research should prioritize disentangling the temporal and causal relationships between depression and dementia to inform targeted preventive and therapeutic strategies.

Recent studies have highlighted the significant impact of demographic factors, such as age, gender, and race, on the prevalence of dementia and depression. For instance, a Danish study investigating female nurses with depression at different life stages found that those who developed depression in late life exhibited a higher risk of progressing to dementia compared to those who experienced depression in middle age (Hickey et al., 2023). Gender differences also play a pivotal role; women have a 55% higher risk of developing dementia than men starting at the age of 65 (Shaw et al., 2021). Similarly, depression disproportionately affects women compared to men, with these gender disparities becoming evident as early as adolescence (Kuehner, 2003). Racial disparities further complicate the clinical landscape. Among patients with comorbid dementia and depression, notable differences were observed in the utilization of antidepressant treatments (Bhattacharjee et al., 2017). Additionally, Asian patients within this cohort demonstrated a lower mortality rate compared to white British patients (Lewis et al., 2018). These findings underscore the importance of considering demographic variables in understanding the complex interplay between dementia and depression.

### 4.3 Research hotspots and development trends

#### 4.3.1 Neuropsychiatric manifestations in dementia and depression

Neuropsychiatric symptoms associated with dementia and depression present a complex challenge, as they significantly impact cognitive functions and emotional regulation in affected individuals. These symptoms can manifest as part of several underlying conditions but are particularly prominent in disorders like MDD and dementia.

##### 4.3.1.1 Bipolar disorder

Bipolar disorder (BD) is a chronic condition that often leads to cognitive impairments affecting memory, attention, and executive function, even during remission phases (Li et al., 2020; Martínez-Arán et al., 2004). These impairments significantly reduce patients’ quality of life and daily functioning (O’Donnell et al., 2017; Sanchez-Moreno et al., 2018). The prevalence of dementia in BD patients ranges from 20 to 25%, notably higher than the 7% observed in the general population over 60 (Almeida et al., 2016; Nunes et al., 2007; Prince et al., 2013). Factors such as antipsychotic treatment, age, and polypharmacy have been identified as predictors of cognitive decline in these patients (Tsapekos et al., 2021). Additionally, cerebral white matter deficits are associated with these cognitive challenges (Macoveanu et al., 2021). Meta-analyses indicate that BD patients have an elevated risk of developing dementia, particularly AD (Velosa et al., 2020; Aprahamian et al., 2013), with each BD episode increasing dementia risk by 6% (Kessing and Andersen, 2004). Research by Knorr et al. (2022) suggests that variations in cerebrospinal fluid markers during emotional relapses, specifically increased a Aβ42 and decreased hyperphosphorylated tau, could contribute to cognitive deterioration and a higher AD risk in BD patients AD related biomarkers in BD—A longitudinal one-year case–control study. Moreover, a study by Xiao Ming revealed that BD patients using valproate have a 59% higher risk of dementia compared to those not using the drug (Moon et al., 2022).

##### 4.3.1.2 Anxiety

Anxiety is commonly observed in dementia and MCI, often coexisting with depression (Hwang et al., 2004; Seignourel et al., 2008). A history of anxiety is linked to a higher risk of all-cause dementia (Kuring et al., 2020). Meta-analysis shows anxiety prevalence in dementia at 38, 41, and 37% across mild, moderate, and severe stages, respectively (Leung et al., 2021), and between 9.9 to 52% in MCI patients (Chan et al., 2011; Gallagher et al., 2011; Lyketsos et al., 2002; Palmer et al., 2007). Studies suggest that anxiety and Aβ interactions accelerate cognitive decline, with anxiety predicting cognitive deterioration in both cognitively unimpaired individuals and MCI patients (Johansson et al., 2020; Pietrzak et al., 2015). Anxiety contributes to the progression from MCI to AD, either directly or through depression (Palmer et al., 2007; Potvin et al., 2011). Biringer et al. (2005) noted that high anxiety impacts cognitive function only alongside depressive symptoms. Contrarily, Devier et al. (2009) argue that state anxiety is not a significant predictor of AD, while higher trait anxiety may lower AD risk. Further long-term studies are necessary to clarify anxiety’s role in dementia progression.

##### 4.3.1.3 Major depressive disorder

Numerous studies indicate that major depression significantly increases the risk of dementia, particularly AD (Diniz et al., 2013). Elderly individuals with MDD, whether in the onset or remission phase, often experience MCI, further elevating their dementia risk (Barnes and Yaffe, 2011; Koenig et al., 2014). The potential connection between major depression and dementia may involve abnormalities in the frontal executive circuit of the executive control network and the corticolimbic circuit of the default mode network, which are thought to diminish cognitive reserve and increase the risk of dementia or AD (Butters et al., 2008). The use of antidepressants in treating MDD has garnered attention regarding its implications for dementia risk. Conflicting results have emerged from various research designs. For example, an Israeli prospective cohort study associated antidepressant use in middle and late life with an increased dementia risk after adjusting for confounders (Kodesh et al., 2019). Conversely, a study involving U.S. veterans found no correlation between antidepressant exposure and long-term dementia risk, regardless of whether antidepressants were used or not (Ramos-Cejudo et al., 2024). The role of MDD in influencing these divergent findings warrants further investigation.

Emotional disorders such as depression, mania, and anxiety are common in prodromal dementia syndrome and often precede cognitive decline. Anxiety is characterized by feelings of nervousness or unease, often without a clear objective cause. MDD primarily presents with persistent low mood, loss of interest, pessimism, cognitive slowing, lack of motivation, poor appetite, and sleep disturbances, with severe cases involving suicidal ideation or behaviors. Bipolar disorder is defined by episodes of extreme mood swings, ranging from depressive lows to hypomanic or manic highs. A survey of 1,399 elderly psychiatric patients with dementia found that 8.3% had bipolar disorder, 10.5% had anxiety disorders, and 12.2% had recurrent depression (Kershenbaum et al., 2021), indicating a higher dementia risk in depression compared to anxiety or bipolar disorder. Additionally, a study on severe mental illness and dementia risk reported that depression independently predicted dementia onset between ages 65–79, while bipolar disorder and anxiety disorders were associated with onset between ages 65–84 (Zilkens et al., 2014).

#### 4.3.2 Mechanisms of dementia and depression

The mechanisms underlying dementia and depression are complex and multifaceted, involving biological, environmental, and psychological factors. Among the biological aspects, recent research has highlighted three critical pathways: the role of the gut microbiome, oxidative stress and functional connectivity.

##### 4.3.2.1 Gut flora

Research over the past 20 years highlights the bidirectional communication of the “gut-brain axis,” through which the intestinal flora regulates the immune, metabolic, and nervous systems. Dysregulation of this axis is implicated in neuropsychiatric diseases, including dementia (Morais et al., 2021; Fan and Pedersen, 2021). Communication occurs via neural pathways, immune regulators, and chemical transmitters (Morais et al., 2021). The intestinal flora connects the gut to organs like the brain, influencing health by altering neuronal circuits, structure, and function through signaling and neurotrophic substances, thereby affecting brain development and behavior (Szabo, 2015; Diaz Heijtz et al., 2011). Recent studies have highlighted the significant role of the gut microbiome in brain function and behavior, particularly concerning dementia and depression. There is compelling evidence that these conditions’ pathogenesis is closely linked to changes in the gut microbiota, where dysbiosis plays a crucial regulatory role (Diaz Heijtz et al., 2011; Goyal et al., 2021; Luna and Foster, 2015). For example, Mosaferi et al. (2021) demonstrated that depleting the gut microbiota in early adolescent mice predisposed to AD could prevent associated anxiety and depressive behaviors. Research also suggests that SSRIs like escitalopram not only improve depressive symptoms in AD patients but may also slow disease progression and reduce the progression from MCI to dementia (Bartels et al., 2018; Dafsari and Jessen, 2020). Notably, escitalopram treatment has been shown to decrease Aβ42 levels in the cerebrospinal fluid of healthy elderly subjects and restore gut flora composition to normal levels (Sheline et al., 2020; Shen et al., 2021). These effects suggest that SSRIs may influence AD and cognitive deterioration partly through their impact on the gut microbiota. Intestinal microbial disturbances promote endotoxins, prostaglandins, and inflammatory cytokines, contributing to neuroinflammation and activating peripheral immune cells (Singh et al., 2016). Immune dysregulation is a key link between depression and cognitive decline (Hayley et al., 2021). Chronic stress accelerates dementia onset in depression via cytokines, with elevated interleukin-6 and tumor necrosis factor-*α* levels observed in the serum, cerebrospinal fluid, and prefrontal cortices of depressed patients (Pandey et al., 2018; Tonelli et al., 2008). Proinflammatory cytokines increase blood–brain barrier permeability, impair serotonin metabolism, and elevate oxidative stress byproducts like quinolinic acid, damaging neurons and glia and leading to cognitive decline (Hayley et al., 2021; Myint and Kim, 2014). Chronic neuroinflammation disrupts synaptic plasticity and neurogenesis, linking serotonin dysregulation to AD pathogenesis (Allison and Ditor, 2014; Caraci et al., 2010). These findings highlight depression as both a risk factor and prodromal symptom of AD (Elsworthy and Aldred, 2019; Caraci et al., 2010).

Additionally, both depression and AD have been linked to an increase in gut bacteria such as Actinobacteria and Ruminococcus, with a notable decrease in *Akkermansia Muciniphila* (Zhao et al., 2022; Zhuang et al., 2018). Enhancing the presence of *Akkermansia Muciniphila* and its secreted extracellular vesicles has shown promise in boosting serotonin signaling and synthesis in the brain and gut, suggesting a therapeutic potential (Yaghoubfar et al., 2020; Yaghoubfar et al., 2021). This understanding of the relationship between serotonin signaling and gut microbiota alterations could be crucial for improving diagnostics and interventions for AD and depression comorbidities. The serotonergic system, notably impaired in early-stage AD, plays a critical role in maintaining cerebral vascular health (Kepe et al., 2006). Serotonergic innervation of brain blood vessels has been shown to regulate cerebral blood flow, which is essential for understanding AD pathophysiology (Colaianna et al., 2010). Studies demonstrate that soluble Aβ injection in rat brains significantly decreases serotonin (5-HT) levels in the prefrontal cortex, a region crucial for mood regulation and highly susceptible to Aβ toxicity. This reduction in 5-HT contributes to depressive symptoms and neurodegeneration in AD (Krishnan and Nestler, 2008; Mattson, 2004; Egashira et al., 2005). Moreover, positron emission tomography (PET) imaging in cognitively normal individuals reveals that enhanced 5-HT signaling reduces Aβ accumulation (Sheline et al., 2014). Pharmacological interventions further highlight the interplay between serotonin and Aβ. Selective COX-2 inhibitors, such as celecoxib, prevent Aβ-induced reductions in 5-HT levels, ameliorating depressive-like behaviors and normalizing Aβ plasma levels (Morgese et al., 2018). In transgenic AD models, SSRIs increase 5-HT signaling, activate extracellular-regulated kinase and *α*-secretase pathways, and decrease Aβ production *in vivo* (Cirrito et al., 2011; Fisher et al., 2016). These findings underscore the potential of targeting serotonergic pathways to modulate cerebral blood flow and Aβ dynamics, offering insights into therapeutic approaches for AD and comorbid depression.

Future research should prioritize targeted interventions modulating the gut-brain axis to address depressive symptoms and cognitive decline in dementia. While SSRIs, probiotics, and dietary modifications show promise in restoring microbiota balance and reducing inflammation, large-scale clinical trials are essential to confirm their efficacy and safety. Developing therapies like *Akkermansia muciniphila* based treatments or fecal microbiota transplantation could offer novel strategies for slowing Alzheimer’s disease progression. Longitudinal studies integrating metagenomics and neuroimaging are needed to clarify the temporal links between microbiota dysbiosis, serotonin signaling, and neurodegeneration, paving the way for personalized treatments.

##### 4.3.2.2 Oxidative stress

Oxidative stress refers to the imbalance between oxidants and antioxidants. When the oxidants are in favor, the redox conduction and control are destroyed or damaged (Sies, 2020). Oxidative stress is a pathogenic mechanism in most neurodegenerative diseases, and neurons are highly sensitive to free radical-mediated damage (Musiek et al., 2013). Research suggests that accumulated oxidative stress is a key mechanism affecting brain aging and the progression of AD (Ionescu-Tucker and Cotman, 2021). The association between oxidative stress and aging has also been found in patients with MDD (Luca et al., 2013) oxidative stress is implicated in disorders such as depression and AD and may serve as a connecting mechanism between them (Black et al., 2015; Butterfield and Mattson, 2020). Notably, depression is often associated with hypercortisolism, which, along with oxidative stress, reduces brain-derived neurotrophic factor expression, leading to hippocampal atrophy (Gillespie and Nemeroff, 2005; O’Brien et al., 2004). This atrophy disrupts the hippocampus’s ability to regulate the HPA axis, exacerbating glucocorticoid levels and further contributing to cognitive decline (Boku et al., 2018; Rubin et al., 2014). Additionally, oxidative stress impairs energy metabolism, particularly in the energy-sensitive hippocampus (Sies, 2015), and promotes inflammation, as evidenced by elevated levels of chemokine motif chemokine ligand 2 (CCL-2) in depression, linked to cognitive deficits and abnormal Aβ metabolism in AD (Romo-Araiza and Ibarra, 2020; Stuart and Baune, 2014; Westin et al., 2012). Moreover, emerging research by Gao et al. (2021) suggests that the α-Klotho gene could link depression and dementia by modulating oxidative stress and inflammation, underscoring its potential role in the neurobiological interplay between these conditions. This highlights the multifaceted impact of oxidative stress in linking depression to dementia, emphasizing the need for further research into its underlying mechanisms and therapeutic targeting.

##### 4.3.2.3 Functional connectivity

Functional connectivity (FC) studies have delineated the impact of neuronal activation patterns and their communication across brain regions, essential for understanding brain function and behavior (Kelly and Castellanos, 2014). Recent research has shown that dementia patients with depressive symptoms (ADD) display reduced FC in the bilateral amygdala and inferior frontal gyrus compared to both patients without depression and healthy controls, indicating a crucial role of the amygdala-frontal circuit in emotional regulation (Du et al., 2024). Furthermore, abnormal locus coeruleus function has been associated with increased FC in the left locus coeruleus and right superior frontal gyrus in ADD patients, underscoring its relationship with depression (Du et al., 2020; Dai et al., 2023). These findings are supported by Ghanbari et al. (2022) analysis, which revealed similar subnetwork changes in AD and MDD, suggesting shared neural substrates.

In parallel, neuroimaging techniques like florbetaben positron emission tomography have provided insights into the brain structural changes due to depression and amyloid deposition, highlighting the significance of early detection in elderly patients with cognitive impairments (Hyung et al., 2021). Studies by Liu et al. further explore how depressive symptoms in MCI patients correlate with specific brain network dysfunctions, particularly noting the role of the left alpha binding cerebral hemisphere and the therapeutic potential of citalopram in improving both cognitive functions and emotional symptoms through distinct neural network interactions (Liu et al., 2022; Diaconescu et al., 2011). Lastly, reduced connectivity between the hypothalamus and temporal regions in AD patients with depression, as identified in a 2018 study, emphasizes the pathophysiological importance of these network disruptions in the manifestation of depressive symptoms within this group (Liu et al., 2018).

The kynurenine pathway, the primary route of tryptophan metabolism in humans, accounts for approximately 95% of total tryptophan metabolism and plays a crucial role in balancing neurotoxicity and neuroprotection (Allison and Ditor, 2014). Tryptophan is converted into kynurenine by indoleamine 2,3-dioxygenase (IDO) and tryptophan 2,3-dioxygenase (TDO) (Oxenkrug, 2010). Disruptions in this pathway are implicated in both AD and major depression. In AD, an imbalance in plasma and cerebrospinal fluid kynurenine metabolites promotes disease progression (Cespedes et al., 2022). In depression, elevated glucocorticoids activate TDO, increasing kynurenine levels and its downstream neurotoxic metabolite, quinolinic acid, which damages hippocampal neurons, overactivates NMDA receptors, and leads to hippocampal atrophy, a hallmark of AD (Allison and Ditor, 2014; Rubin, 1967; Wolkowitz et al., 2009). These findings suggest that dysregulation of the kynurenine pathway is a shared pathogenic mechanism contributing to the progression of both disorders. Recent studies reveal a link between Aβ pathology and depressive symptoms in dementia. Aβ oligomers induce depressive-like behaviors in mice, which are alleviated by antidepressants, indicating a direct impact on mood regulation (Kosel et al., 2020; dos Santos et al., 2013; Ledo et al., 2013). During the COVID-19 lockdown, amyloid-positive individuals experienced more severe depressive symptoms under stress, suggesting AD pathology exacerbates depressive responses (Lewis et al., 2022). Patients with mild depressive symptoms also show elevated cerebrospinal fluid amyloid markers and an 83% higher risk of developing AD (Xu et al., 2021). Tau protein, a key factor in neurofibrillary tangles, further connects depression and AD. AD patients with depression show increased tangles due to tau hyperphosphorylation (Rapp et al., 2008). However, a longitudinal study of nearly 2,000 participants did not find a consistent association, reflecting the complexity of tau’s role in depression and AD progression (Wilson et al., 2016).

#### 4.3.3 Management of dementia and depression

##### 4.3.3.1 Non-drug treatment

In the context of the side effects and limited efficacy of psychotropic drugs, there has been a rising interest in non-pharmacological interventions for managing dementia and depression. Music therapy, recognized as a potentially cost-effective non-drug treatment, has demonstrated efficacy in reducing depressive symptoms among dementia patients. Notably, a randomized controlled trial in Australia reported that group music therapy and recreational choir singing significantly alleviated depressive symptoms over a six-month period (Baker et al., 2022). Further, a specific intervention involving reminiscence music therapy sessions twice a week for 4 weeks markedly reduced depressive symptoms, although it showed no significant impact on cognitive and behavioral symptoms (Tz-Han et al., 2023). A recent meta-analysis found that active music therapy combined with singing significantly improves depressive symptoms in dementia patients, outperforming music listening alone (Ting et al., 2024). Group music therapy, by enhancing social interaction and reducing loneliness, is more effective than individual therapy. Additionally, repetitive transcranial magnetic stimulation (rTMS) using intermittent theta burst stimulation (iTBS) has shown promise in improving mood and cognition in patients with dementia and depression (Jin et al., 2024). Xu et al. (2024) study further supported these findings, showing that group music therapy not only lowered depression and salivary cortisol levels but also maintained these reduced levels during a 3-month follow-up. A trial in Australia found that receptive music therapy (RCS) was more effective than group music therapy (GMT) in reducing depressive symptoms in dementia patients and showed benefits for advanced dementia (Baker et al., 2022). Another study demonstrated that music activities designed by music therapists and led by trained nursing assistants reduced depressive symptoms and improved well-being in dementia patients (Ray and Götell, 2018). These findings highlight the potential of continuous music therapy and music-based interventions.

On another front, reminiscence therapy, which uses cues like photos and music to trigger memory recall, has also been proven effective. A 2015 meta-analysis indicated that reminiscence therapy modestly improves cognitive function and significantly ameliorates depression in dementia patients (Huang et al., 2015). Li et al. (2020). found that group reminiscence therapy effectively improved depressive symptoms and neuropsychiatric symptoms in patients with mild to moderate AD. However, a 2021 meta-analysis suggested that while reminiscence therapy shows some potential benefits for enhancing quality of life, its effectiveness in reducing depressive symptoms still requires further investigation (Thomas and Sezgin, 2021). Cognitive psychotherapy is widely used in clinical practice and has shown effectiveness in treating dementia with depressive symptoms. A meta-analysis found that adding cognitive behavioral therapy (CBT) to usual care reduces depressive symptoms and increases remission rates in dementia patients (Orgeta et al., 2022). Mindfulness-based cognitive therapy has also been shown to alleviate depression and anxiety in dementia patients (Douglas et al., 2022), though a randomized controlled trial reported no significant improvement in cognition, anxiety, or depression following mindfulness interventions in mild dementia (Noone et al., 2023). Psychotherapy holds potential for enriching treatment options for dementia with depression, though further evidence is needed. Additionally, traditional Chinese medicine, such as acupuncture, has been shown to improve insomnia and depression by stimulating specific points to regulate brain function (Zhang et al., 2024). Its potential benefits on mood and cognition in dementia patients with depression warrant further exploration, offering a novel direction for future treatments.

##### 4.3.3.2 Drug treatment

In clinical practice, while non-drug treatments can help alleviate depression in dementia patients, pharmacotherapy is essential, particularly for severe cases. Antidepressant therapy is the first-line treatment for dementia patients with depressive symptoms. A meta-analysis of 25 studies indicated that mirtazapine and sertraline are slightly more effective than placebo in managing depressive symptoms in these patients, although clomipramine was associated with an increased risk of adverse events (He et al., 2021). Observational studies have shown that vortioxetine is effective and well-tolerated in patients with prodromal and mild to moderate AD who also suffer from depression, significantly improving cognitive function in those with prodromal AD (Padovani et al., 2024). However, a recent meta-analysis involving eight studies on SSRIs, SSNRIs, tricyclic antidepressants, and atypical antidepressants found no strong evidence that antidepressant treatment improves depression in dementia patients (SMD −0.10 [−0.26, 0.07]) (Lenouvel et al., 2024). These discrepancies may stem from differences in the severity of dementia among study subjects. More accurate screening tools and diagnostic criteria are needed to further clarify the efficacy of antidepressants in this patient population. SSRIs such as sertraline, fluoxetine, paroxetine, citalopram, and escitalopram are widely used to treat depression in AD patients. Long-term SSRI use in individuals with MCI and a history of depression has been shown to delay AD progression by approximately 3 years (Bartels et al., 2018). Escitalopram reduces cerebrospinal fluid Aβ42 levels in cognitively normal older adults (Sheline et al., 2020), and fluoxetine improves spatial learning deficits in AD models by preventing neuronal damage (Ma et al., 2017). These findings suggest that SSRIs may benefit both depressive symptoms and AD progression. However, further studies are needed to clarify the differential effects of specific SSRIs.

Early intervention plays a critical role in both non-pharmacological and pharmacological approaches to managing dementia and depression. Non-drug treatments, such as music therapy and cognitive psychotherapy, have shown potential in early stages to improve mood, enhance social interaction, and possibly delay cognitive decline. For example, group music therapy and reminiscence therapy can be particularly effective in MCI or early AD, providing a non-invasive way to mitigate depressive symptoms and support cognitive health. On the pharmacological front, long-term use of SSRIs in patients with MCI and a history of depression has been shown to delay the progression to AD by up to 3 years. These findings underscore the importance of identifying at-risk individuals early and implementing timely interventions to improve clinical outcomes and slow disease progression.

## 5 Limitations

Although this bibliometric analysis adheres to standard methods and utilizes a robust dataset from the WoSCC database, it has several limitations that may affect the comprehensiveness of the findings. Firstly, the reliance on a single database excludes potentially relevant studies indexed in other platforms like PubMed, Scopus, or Google Scholar. Secondly, bibliometric methods primarily evaluate research impact based on citation counts, which may overlook high-quality but less cited studies. Additionally, there is also an inherent time lag in bibliometrics, which can delay the recognition of emerging research trends.

## 6 Conclusion

This bibliometric analysis of publications on dementia and depression from 2005 to 2024 highlights the leading role of the United States in both research volume and impact, with significant contributions from China. The University of Toronto and notable researchers like Herrmann N and Aarsland D have driven advancements in understanding the neurobiological underpinnings of these conditions. Current research hotspots include the comorbid mechanisms between dementia and depression, such as gut microbiota, oxidative stress, and FC. Additionally, prevalent neuropsychiatric symptoms like anxiety and BD are prominently featured in the literature. The study also anticipates that non-pharmacological interventions will become a crucial area of future research. These insights provide a solid foundation for advancing the understanding and treatment of dementia and depression.

## Data Availability

Publicly available datasets were analyzed in this study. This data can be found at: https://www.webofscience.com/wos/woscc/basic-search.

## Footnotes

^1^ https://bibliometric.com/
